# Case Report: Identification of a Novel Homozygous Mutation in *GPD1* Gene of a Chinese Child With Transient Infantile Hypertriglyceridemia

**DOI:** 10.3389/fgene.2021.726116

**Published:** 2021-08-18

**Authors:** Haihua Lin, Youhong Fang, Lin Han, Jie Chen, Jingan Lou, Jindan Yu

**Affiliations:** ^1^Department of Gastroenterology, Zhejiang University School of Medicine Children's Hospital, National Center for Clinical Medical Research in Children Health and Disease, National Regional Medical Centre for Children, Hangzhou, China; ^2^Running Gene Inc., Beijing, China

**Keywords:** glycerol-3-phosphate dehydrogenase 1, transient infantile hypertriglyceridemia, hepatomegaly, hypohepatia, hepatic steatosis, next-generation sequencing

## Abstract

Transient infantile hypertriglyceridemia is a rare autosomal recessive disorder characterized by hypertriglyceridemia, hypohepatia, hepatomegaly, hepatic steatosis and fibrosis in infancy. Mutations in *GPD1* gene are considered the causative factor but the underlying mechanism of this disorder is still enigmatic. To date, only 24 different *GPD1* mutations have been reported in the literature worldwide with transient infantile hypertriglyceridemia or relevant conditions. Here we report a Chinese girl who developed hepatomegaly hepatic steatosis, elevated transaminase and hypertriglyceridemia from the age of 4 months. A novel homozygous variant c.454C>T (p.Q152^*^) was found in *GPD1* gene by next-generation sequencing. This patient is the 3rd Asian reported with transient infantile hypertriglyceridemia. We summarized the clinical presentations of transient infantile hypertriglyceridemia and also expanded the spectrum of disease-causing mutations in *GPD1*.

## Introduction

Transient infantile hypertriglyceridemia (HTGTI; OMIM#614480) is a rare disorder with an autosomal recessive pattern of inheritance. Most patients with HTGTI present with transient hypertriglyceridemia, elevated transaminases, early-onset hepatomegaly, persistent fatty liver and hepatic fibrosis. Rare phenotypes include fasting hypoglycemia, kidney disease, obesity, insulin resistance, dermal abnormalities and short stature (Dionisi-Vici et al., 2016; Li et al., 2017). This condition has been considered to be caused by mutations in the *GPD1* gene (OMIM#138420) which encodes the intracellular isomer of glycerol-3-phosphate dehydrogenase 1 (GPD1). A total of 31 mutations in the *GPD1* gene have been reported in Human Gene Mutation Database (HGMD) (Stenson et al., 2017), of which only 24 mutations have been described with HTGTI or associated phenotypes worldwide (Basel-Vanagaite et al., 2012; Joshi et al., 2014; Di Resta et al., 2015; Dionisi-Vici et al., 2016; Dron et al., 2017, 2020; Li et al., 2017, 2018; D'erasmo et al., 2019; Matarazzo et al., 2020). Herein we report a Chinese girl with a hitherto unreported homozygous variant of the *GPD1* gene, who presented with elevated transaminases, massive hepatomegaly, hepatic steatosis and hypertriglyceridemia from the age of 4 months.

## Case Presentation

**Figure 1:**
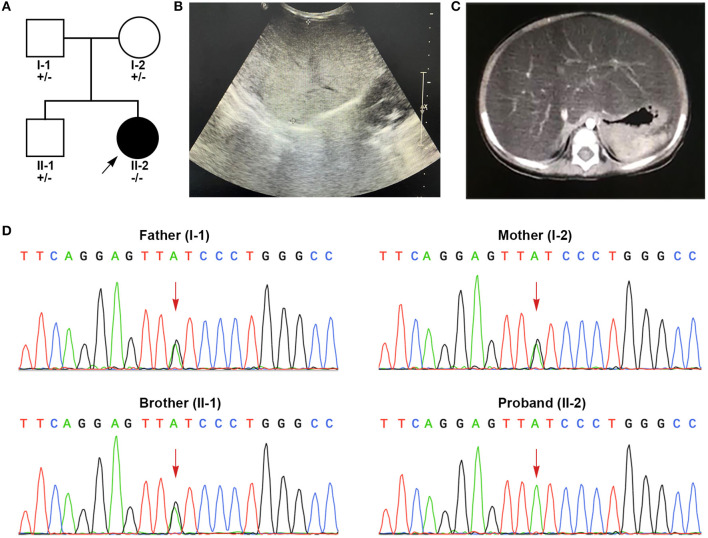
Clinical features and genetic sequencing data of the patient. **(A)** The pedigree of the family. **(B)** B-mode ultrasound demonstrated diffuse hyperechogenicity of the liver with a fine granular pattern. **(C)** Contrast-enhanced CT scan of abdomen shows a significantly enlarged liver with fatty change. **(D)** Sanger sequencing shows that the patient harbored homozygous mutations (c.454C>T, p.Q152*) in exon 6. The patient's parents and brother were all heterozygous for this mutation.

The patient is a 4-year-old Chinese girl born via cesarean section without remarkable complications. Her weight at birth was 3.15 kg (50th percentile) and her height was 50 cm (50th percentile). Her parents were both healthy and non-consanguineous. Her older brother was physically healthy (Figure 1A). The patient has no relevant family history of the disease. No liver dysfunction, hyperlipidemia, or hepatic steatosis has been found in the patient's parents or brother.

The patient suffered from bronchopneumonia at the age of 4 months and 5 days. During this period, liver dysfunction was noticed. After recovering from bronchopneumonia, the patient was referred to our hospital for further examination and treatment due to persistent elevations in liver transaminase. At her physical examination at the age of 4 months, her weight was 5.8 kg (6th percentile) and her height was 61.2 cm (17th percentile). She presented with marked hepatomegaly but splenomegaly. She showed normal growth and psychomotor development. In laboratorial examinations, her results of liver function tests were abnormal. Her alanine aminotransferase (ALT, 107 U/L, reference range (r.r): 0–60 U/L), aspartate aminotransferase (AST, 186 U/L, r.r: 0–60 U/L), γ-glutamyl transpeptidase (γ-GT, 265 U/L, r.r: 0–50 U/L) and total bile acids (18.2 μmol/L, r.r: 0–10 μmol/L) were highly elevated. Blood lipid test also showed elevated triglycerides (TG, 4.39 mmol/L, r.r <1.7 mmol/L). The levels of total cholesterol, lipoprotein, bilirubin, coagulation test and albumin were within normal limits. Further workups including routine blood test, blood glucose, electrolytes, serum ammonia, lactic acid levels, fatty acids, ceruloplasmin, serological markers for viruses (cytomegalovirus, Epstein-Barr virus, hepatitis virus), toxoplasmosis, thyroid function, creatinine, uric acid, autoimmune serology and immunoglobulin levels, all of which showed normal results. B-mode ultrasound of the liver showed diffuse hepatomegaly with increased echogenicity (Figure 1B). Contrast-enhanced abdominal CT suggested significant hepatomegaly with fatty changes (Figure 1C). No splenomegaly was observed. The results of echocardiogram showed atrial septal interruption (Φ 0.31 cm). No abnormalities were observed in electrocardiogram (ECG), chest radiographs, portal vein and inferior vena cava ultrasonography. Liver biopsy was recommended but her parents refused.

After administrating compound glycyrrhizin tablets (half tablet per day) (Each tablet contains 25 mg glyeyrrhizin, 35 mg monoammonium glycyrrhizinate, 25 mg aminoacetic acid and 25 mg methionine; Minophagen Pharmaceutical Co., Ltd., Japan) for 3 months, her transaminase levels decreased but remained above normal (ALT, 85 U/L; AST, 127 U/L; γ-GT, 258 U/L.). At 3.5 years old, her TG and transaminases were at normal levels and her abdominal ultrasound showed that her liver was smaller in size than before. At the age of 4, her weight was 13 kg (3rd percentile) and her height was 94 cm (3rd percentile). Except for short stature, no other symptoms were presented.

## Genetic Analysis

The patient was suspected with hereditary metabolic liver disease due to a combination of elevated liver transaminases, hypertriglyceridemia and hepatomegaly. Peripheral blood samples of the patient, her parents and her brother were collected and sent to Running Gene Inc. (Beijing, China) for next-generation sequencing (NGS). DNA samples were extracted using the QIAamp DNA Blood Midi Kit (Qiagen, Hilden, Germany) and quantified using Nanodrop spectral analysis (Thermo Fischer Scientific, Inc., Waltham, MA). The patient's genomic DNA was fragmented into 200–300 bp for sequencing library generation by KAPA Library Preparation Kit (#KR0453, Kapa Biosystems, Wilmington, MA). Pooled libraries were screened by the customized xGen Inherited-Diseases-Panel Probe. The captured libraries were sequenced by the Illumina hiseq2500 Analyzers (Illumina, San Diego, CA). Single-nucleotide variants and indels were called and filtered based on multiple databases [ExAC (Lek et al., 2016), gnomAD (Karczewski et al., 2020), 1kGenome (Genomes Project et al., 2015), ESP6000 (Fu et al., 2013), ClinVar (Landrum and Kattman, 2018), and HGMD]. Candidate variants were classified according to the American College of Medical Genetics and Genomics (ACMG) guidelines (Richards et al., 2015). Suspected pathogenic variants were then verified in the proband and her parents by Sanger sequencing.

**Figure 2:**
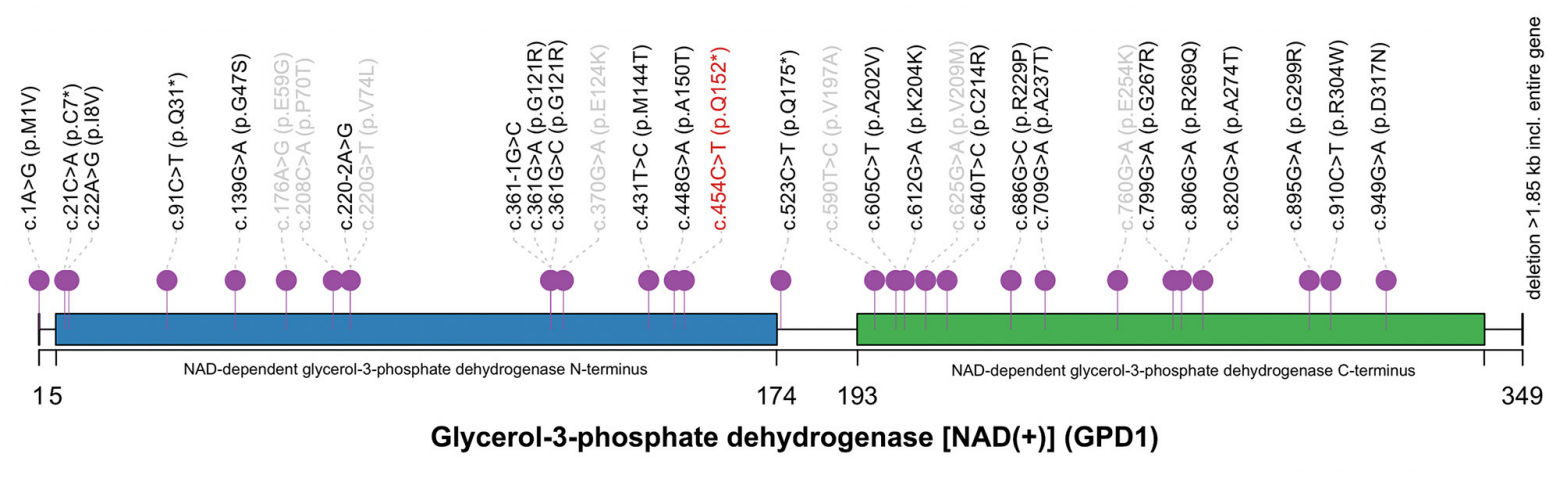
The spectrum of *GPD1* mutations reported in HGMD database. The novel variant c.454C>T (p.Q152*) (red) located in NAD-dependent glycerol-3-phosphoate dehydrogenase N-terminus. Twenty-four mutations relevant to HTGTI are marked in black. Seven mutations not associated with HTGTI are labeled in gray. This spectrum was generated by R with trackViewer: lollipopPlot (Ou and Zhu, 2019).

A pair of homozygous non-sense variant c.454C>T (p.Q152^*^) was identified in the *GPD1* gene (NM_001257199). The mutated variants of the proband were inherited from her parents, in fact, the same variant was also found in heterozygosis in her parents and her brother, consistent with the pattern of recessive inheritance (Figure 1D). This variant, which has not been recorded in any public databases, is novel. Variant c.454C>T altered codon for Glu152 to a termination codon, which may produce a truncated protein or lead to non-sense-mediated mRNA decay (PVS1). This variant is located in N-terminal NAD-dependent glycerol-3-phosphate dehydrogenase (Figure 2) (PM1) and absent from controls (ExAC, gnomAD, 1kGenome, ESP6000) (PM2). Multiple software predicted the variant as deleterious [MutationTaster2 (Schwarz et al., 2014), 1.000, disease causing; Ba+Del_addAF (Feng, 2017), 0.625 > cutoff = 0.069, deleterious; CADD (Rentzsch et al., 2019), 35 > 15, damaging; fathmmMKL-inherited (Shihab et al., 2013), 0.927 > 0.5, damaging.] (PP3). Thus, c.454C>T (p.Q152^*^) is classified as a pathogenic variant (PVS1+PM1+PM2+PP3), according to the ACMG guidelines.

## Discussion

*GPD1* mutation related to HTGTI was first discovered by Basel-Vanagaite et al. in 10 family members of four highly consanguine Israeli Arab families carrying c.361-1G>C (Basel-Vanagaite et al., 2012). Subsequently, nine articles described extra 30 mutations of *GPD1* gene associated with HTGTI, chylomicronemia syndrome, Brugada syndrome or low HDL cholesterol, respectively. Only six articles worldwide described complete clinical presentation and genetic data of 18 patients with HTGTI or relevant phenotypes, and we summarized them with our current patient (Table 1). The mean age of onset was about 10 months, with a median age of 5 months. The gender ratio (male to female) is 1.375 (11:8). Common clinical features of *GPD1*-associated patients are hepatic steatosis (100%, 19/19), hepatomegaly (100%, 18/18), hypertriglyceridemia (95%, 18/19), and hypohepatia (elevated liver transaminases) (95%, 18/19). Other phenotypes, including splenomegaly (4/11), fibrosis (6/19), short stature (6/19), vomiting (4/19), elevated total bile acids (4/19), and failure to thrive (3/19), are also involved. The affected individuals tended to vomit and fail to thrive as the first symptoms, but more than half of cases were asymptomatic. A third of patients had a liver biopsy, showing steatosis with varying degrees of fibrosis. Our patient has characteristics of *GPD1* deficiency includes early-onset, elevated ALT and γ-GT levels, hypertriglyceridemia, fatty liver and mild failure to thrive. The clinical manifestations were similar to the 18 patients described in the previous articles (Basel-Vanagaite et al., 2012; Joshi et al., 2014; Dionisi-Vici et al., 2016; Li et al., 2017, 2018; Matarazzo et al., 2020). Although phenotypic studies of *GPD1* mutations have focused on dyslipidemia and secondary hepatic metabolic disorders, the clinical features of patients reported by Dionisi-Vici et al. were slightly different from other reported cases. One patient had recurrent fasting hypoglycemia; the second presented with severe liver disease with intrahepatic cholestasis involving the kidney. A patient with rare phenotypes (obesity, insulin resistance, pimple, acanthosis nigricans, hypertrichosis and short stature) was also reported (Li et al., 2017). Based on the available case reports, the severity of the clinical phenotype of *GPD1* mutation-associated HTGTI is independent of the type and location of the mutation site.

**Table 1:** Clinical and genetic characteristics of *GPD1*-associated 19 HTGTI patients whose data are available.

**References**	**Case**	**Onset age (m)**	**Gender (M:F, 11:8)**	**Mutations in *GPD1* gene**	**Hypertriglyceridemia (Elevated TG) (18/19)**	**Last TG**	**Hypohepatia (Elevated transaminases) (18/19)**	**Last transaminases**	**Hepatomegaly (18/18)**	**Splenomegaly (4/11)**	**Hepatic steatosis (19/19)**	**Other features**
Present	1	4	F	c.454C>T, p.Q152*	+	–	+	–	+	–	+	TBA elevation; Failure to thrive; Short stature
Li et al. (2017)	2	84	M	c.220-2A>G;c.820G>A, p.A274T	–	–	–	–	NA	NA	+	Short stature; Obesity; Insulin resistance; Dermal abnormalities; EDL
Basel-Vanagaite et al. (2012)	3	1	M	c.361-1G>C, p.I119fs*94	+	↑	+	–	+	NA	+	Vomiting
Basel-Vanagaite et al. (2012)	4	1	M	c.361-1G>C, p.I119fs*94	+	↑	+	↑	+	NA	+	Vomiting; Hepatic fibrosis;
Basel-Vanagaite et al. (2012)	5	4–6	M	c.361-1G>C, p.I119fs*94	+	↑	+	–	+	–	+	Short stature; Hepatic fibrosis
Basel-Vanagaite et al. (2012)	6	At birth	M	c.361-1G>C, p.I119fs*94	+	–	+	↑	+	+	+	Short stature
Basel-Vanagaite et al. (2012)	7	6	F	c.361-1G>C, p.I119fs*94	+	↑	+	↑	+	NA	+	Failure to thrive
Basel-Vanagaite et al. (2012)	8	2.5	F	c.361-1G>C, p.I119fs*94	+	–	+	–	+	–	+	Vomiting
Basel-Vanagaite et al. (2012)	9	7	F	c.361-1G>C, p.I119fs*94	+	–	+	↑	+	+	+	-
Basel-Vanagaite et al. (2012)	10	7	F	c.361-1G>C, p.I119fs*94	+	↑	+	↑	+	+	+	-
Basel-Vanagaite et al. (2012)	11	9	M	c.361-1G>C, p.I119fs*94	+	↑	+	↑	+	–	+	Short stature; Horseshoe kidney
Basel-Vanagaite et al. (2012)	12	3.5	M	c.361-1G>C, p.I119fs*94	+	↑	+	↑	+	–	+	Short stature; Craniocerebral involvement
Dionisi-Vici et al. (2016)	13	12	F	c.361-1G>C, p.I119fs*94	+	–	+	NA	+	NA	+	-
Li et al. (2018)	14	3.5	F	c.523C>T, p.Q175*	+	↑	+	↑	+	-	+	TBA elevation; Portal fibrosis
Dionisi-Vici et al. (2016)	15	5	M	c.640T>C, p.C214R	+	↑	+	↑	+	+	+	Consanguineous parents; TBA elevation; Cirrhosis; Dicarboxylic aciduria
Dionisi-Vici et al. (2016)	16	24	M	c.640T>C, p.C214R	+	↑	+	NA	+	NA	+	Portal and periportal Fibrosis
Joshi et al. (2014)	17	At birth	F	Deletion >1.85 kb including entire *GPD1*; c.686G>C, p.R229P	+	↑	+	NA	+	NA	+	Small head circumference; Failure to thrive; Vomiting
Dionisi-Vici et al. (2016)	18	10	M	c.806G>A, p.R269Q	+	–	+	↑	+	NA	+	Consanguineous parents; Fasting hypoglycemia; Portal and bridging fibrosis
Matarazzo et al. (2020)	19	12	M	c.895G>A, p.G299R	+	–	+	↑	+	–	+	TBA elevation

*EDL, elevated dehydroepiandrosterone sulfate and lipoprotein-α levels; NA, not available; TBA, total bile acid; TG, triglyceride; m, months; y, years*.

Although hypertriglyceridemia is a significant risk factor for coronary disease, acute pancreatitis and metabolic syndrome (Cullen, 2000; Athyros et al., 2002; Hopkins et al., 2005), all affected individuals had a relatively good medium-term prognosis. According to follow-up data from patients with *GPD1* mutations, in most patients, the TG and liver enzyme indices normalize with age and no specific treatment is required. Furthermore, no clinical evidence of coronary disease or pancreatitis has been reported, nor in the oldest patients (Basel-Vanagaite et al., 2012). Therefore, liver transplant is not recommended for patients with HTGTI. Lipid-lowering drugs are not routinely used because TG levels can be ameliorated without particular treatment. That proves the diagnosis of *GPD1* deficiency is of great significance for the management of the patients. Although no conclusions can be drawn about the risks in adulthood or long-term prognosis, sufficient attention must be given to HTGTI patients during long-term follow-up. Assessment of the growth and development and screening for abnormal indicators such as liver transaminases, total cholesterol, TG, abdominal ultrasound are recommended at follow-up visits.

Located on chromosome 12q13.12, *GPD1* gene encodes glycerol-3-phosphate dehydrogenase 1, a member of the NAD-dependent GPD family. GPD1 is a protein of 37.5 kD size that catalyzes the invertible conversion of dihydroxyacetone phosphate (DHAP) and nicotine adenine dinucleotide (NADH) to glycerol-3-phosphate (G3P) and NAD+ in the cytoplasm, working in the metabolism of carbohydrate and lipid (Menaya et al., 1995; Ou et al., 2006; Basel-Vanagaite et al., 2012). Along with GPD2 (mitochondrial), GPD1 (cytosolic) also forms a glycerol phosphate shuttle which plays an important role in the transport of reducing equivalents from the cytosol to mitochondria, resulting in the reoxidation of NADH formed from glycolysis and the regeneration of NAD+ in the brain and skeletal muscle.

A total of 31 mutations of *GPD1* were reported in HGMD, including 24 mutations associated with HTGTI or relative phenotypes. Of these 24 mutations, only 11 mutations are disease-causing (DM) and 13 are possibly disease-causing (DM?). Including our current novel variant, only four non-sense have been reported in *GPD1* and they are all classified as DM. However, the underlying mechanism of HTGTI caused by *GPD1* mutations is still unknown. Only a few theories have been hypothesized and studied. Since the availability of G3P has been considered as a regulatory factor of TG synthesis, it has been suggested that *GPD1* mutations may cause an increase in the amount of hepatic G3P available for TG synthesis by limiting the conversion of G3P to DHAP, consequently leading to elevated TG (Basel-Vanagaite et al., 2012). Intriguingly, as the conversion is from DHAP to G3P under physiological conditions, it is also possible that DHAP may not be converted to G3P due to the lack of *GPD1*, resulting in a relative surplus of DHAP and a decrease in G3P. This theory has been demonstrated in a mouse model of *GPD1* deficiency (Macdonald and Marshall, 2000). DHAP can be acylated firstly then reduced to 1-acyl-sn-G3P. The acyl DHAP pathway also plays a significant role in the synthesis of glycerides in the liver (Athenstaedt and Daum, 2000; Zheng and Zou, 2001). The pathogenesis of fatty liver in HTGTI patients may be caused by excessive DHAP acylation. *GPD1* mutations lead to increased TG synthesis in the liver, decreased output from the liver, increased inflow of fatty acids into the liver, and impaired hepatic beta-oxidation causing non-alcoholic hepatic steatosis (NASH) (Dionisi-Vici et al., 2016). In two of the studies, *in vitro* functional studies using cell experiments confirmed the pathogenicity of the c.361-1G>C, c.220-2A>G and c.820G>A mutations (Basel-Vanagaite et al., 2012; Li et al., 2017). Only one of the affected individuals described in the above studies has an overweight body mass index, which refutes the role of obesity in the pathophysiology of the development of hepatic steatosis in these individuals. The pathogenesis of each symptom in *GPD1*-associated HTGTI requires more clinical and basic medical research to be explored.

In summary, this study reported a Chinese HTGTI patient and also expanded the spectrum of disease-causing mutations in *GPD1* gene. In future clinical diagnosis, *GPD1* deficiency should be considered if hypertriglyceridemia, elevated liver enzymes, hepatomegaly, hepatic steatosis and fibrosis are present in early infancy. When the cause cannot be determined by traditional clinical examination, NGS is a promising approach to help clinical diagnosis and guide clinical management.

## Data Availability Statement

The original contributions presented in the study are included in the article/supplementary materials, further inquiries can be directed to the corresponding author. Genetic sequencing data are available in Sequence Read Archive (SRA) by PRJNA747113.

## Ethics Statement

This study was reviewed and approved by the Research Ethics Committee of The Children's Hospital, Zhejiang University School of Medicine (2020-IRB-166, November 16, 2020). Written informed consent was obtained from the patient's parents for the investigation and publication of this article.

## Author Contributions

HL analyzed and interpreted the patient data and drafted the manuscript. JC and JL were responsible for guiding the diagnosis and treatment of the patient. LH analyzed sequencing data, produced figures, and made a significant contribution to the manuscript. YF contributed to gathering patient data and was a major contributor in writing the manuscript. HL and LH reviewed the final manuscript. All authors read and approved the final manuscript.

## Conflict of Interest

LH is employed by Running Gene Inc., Beijing, China. The remaining authors declare that the research was conducted in the absence of any commercial or financial relationships that could be construed as a potential conflict of interest.

## Publisher's Note

All claims expressed in this article are solely those of the authors and do not necessarily represent those of their affiliated organizations, or those of the publisher, the editors and the reviewers. Any product that may be evaluated in this article, or claim that may be made by its manufacturer, is not guaranteed or endorsed by the publisher.
